# External Validation of a Prognostic Model for Seizure Recurrence Following a First Unprovoked Seizure and Implications for Driving

**DOI:** 10.1371/journal.pone.0099063

**Published:** 2014-06-11

**Authors:** Laura Jayne Bonnett, Anthony G. Marson, Anthony Johnson, Lois Kim, Josemir W. Sander, Nicholas Lawn, Ettore Beghi, Maurizio Leone, Catrin Tudur Smith

**Affiliations:** 1 Department of Biostatistics, University of Liverpool, Liverpool, United Kingdom; 2 Department of Molecular and Clinical Pharmacology, University of Liverpool, Liverpool, United Kingdom; 3 Medical Research Council Biostatistics Unit, Cambridge Institute of Public Health, Cambridge, United Kingdom; 4 Department of Medical Statistics, London School of Hygiene and Tropical Medicine, London, United Kingdom; 5 National Institute for Health Research, University College London Hospitals Biomedical Research Centre, London, United Kingdom; 6 Western Australian Comprehensive Epilepsy Centre, Royal Perth and Fremantle Hospitals, Perth, Australia; 7 Department of Neuroscience, Istituto di Ricerche Farmacologiche Mario Negri, Milano, Italy; 8 Clinica Neurologica, Ospedale Maggiore della Carità, Novara, Italy; 9 Medical Research Council Clinical Trials Unit, London, United Kingdom; 10 University College London Institute of Neurology, London, United Kingdom; 11 Epilepsy Society, Chalfont St Peter, United Kingdom; University of Louisville, United States of America

## Abstract

**Objective:**

In the United Kingdom and other European Union countries guidelines for driving following a first unprovoked seizure require the risk of another seizure in the next year to be less than 20%. Using data from one clinical trial, we previously developed a prognostic model to inform driving guidelines. The objective of this work is to externally validate our published model and demonstrate its generalisability.

**Methods:**

A cohort of 620 people with a first unprovoked seizure was used to develop the original model which included variables for aetiology, first degree relative with epilepsy, seizures only while asleep, electroencephalogram, computed tomography or magnetic resonance scan result, and treatment policy. The validation cohorts consisted of 274 (United Kingdom), 305 (Italy), and 847 (Australia) people. The model was evaluated using discrimination and calibration methods. A covariate, missing from the Italian dataset, was handled via five imputation methods. Following external validation, the model was fitted to a pooled population comprising all validation datasets and the development dataset. The model was stratified by dataset.

**Results:**

The model generalised relatively well. All methods of imputation performed fairly similarly. At six months, the risk of a seizure recurrence following a first ever seizure, based on the pooled datasets, is 15% (95% CI: (12% to 18%)) for patients who are treated immediately and 18% (95% CI: (15 to 21%)) otherwise. Individuals can be reliably stratified into risk groups according to the clinical factors included in the model.

**Significance:**

Our prognostic model, used to inform driving regulations, has been validated and consequently has been proven as a valuable tool for predicting risk of seizure recurrence following a first seizure in people with various combinations of risk factors. Additionally, there is evidence to support one worldwide overall prognostic model for risk of second seizure following a first.

## Introduction

In the UK and other EU countries, following a first unprovoked seizure, the majority of people are allowed to return to driving a car (group 1 license holders) once they have gone six months without a seizure - by this time point their risk of a subsequent seizure in the next 12 months is estimated to have dropped below 20%. This recommendation is in part informed by prognostic modelling of data from the Multicentre Study of Early Epilepsy and Single Seizures (MESS) [1], [2]. A prognostic model was also developed [3] aiming to determine the overall population risk of a seizure recurrence in the next 12 months at differing time points after a first seizure, and to identify which clinical factors influenced seizure recurrence risk. This allowed people with a first seizure to be stratified according to the risk of seizure recurrence, including those with a 12 month recurrence risk below 20% and those with a risk above 20% six months after a first seizure. The model included variables for aetiology, epilepsy in a first degree relative, seizure while asleep, electroencephalogram (EEG) results, computed tomography or magnetic resonance (CT/MRI) imaging scan results, and treatment policy [3].

Before a predictive or prognostic model can be introduced into routine practice, it should be externally validated to ensure it performs satisfactorily in datasets that are fully independent of the development data [4]. The datasets used to externally validate the model should be plausibly related to the development data meaning that all datasets will effectively be samples taken from the same super-population [4]. In the case of MESS there are several plausibly related datasets for which we have individual patient data: (1) The UK-based National General Practice Study of Epilepsy (NGPSE) [5], (2) data prospectively collected at hospital-based epilepsy clinics in Perth, Western Australia (WA) [6], [7], and (3) the FIRST (FIRST) dataset from Italy [8]–[10].

We describe the external validation of the model from MESS in these external datasets. External validation of our prognostic model demonstrated that it is a valuable tool for predicting risk of seizure recurrence following a first seizure in people with various combinations of risk factors. Additionally, we fit the prognostic model to a combined dataset of MESS, NGPSE, WA and FIRST. A good model fit to the dataset provided support for one worldwide overall prognostic model for risk of second seizure following a first which will enable driving regulations worldwide to be harmonised.

## Methods

### Description of Studies

The data for each study used in these analyses are available from representatives of the original studies – these are listed as co-authors.

#### Study used for model development

MESS [1] was a UK-based randomised controlled trial that compared the policies of immediate or deferred treatment in people presenting with a first unprovoked seizure, or with epilepsy, where both the clinician and patient were uncertain about the need for antiepileptic drug (AED) treatment. MESS remains the largest reported study of people with single seizures and early epilepsy, and while the primary purpose of the study was to compare treatment policies, it also provided an important opportunity to examine seizure recurrence risks and factors that modify those risks.

#### Studies used for validation

The NGPSE [5] was initiated in 1984 and used the UK primary care system to obtain comprehensive data on a large and unselected cohort of people with newly diagnosed seizures, including children with febrile seizures. Patients were ascertained for the study via their general practitioner (GP), but were never contacted directly by the study team. Accepted practice at the time for observational studies, was that individuals did not need to be asked for consent as their care was never affected by inclusion in such studies. Over a thousand people were initially referred by their GPs, of who a quarter were children with febrile seizures. About two thirds had definite or probable epilepsy and seizure dates were ascertained retrospectively in a proportion.

The WA [6], [7] dataset included adults referred to the First Seizure Clinics of Royal Perth and Fremantle Hospitals, two major teaching hospitals in Western Australia. Recruitment began in January 1999 and is on-going. The data presented here represent information collected up until March 2011.

The FIRST [8]–[10] dataset comprises participants from a randomised clinical trial that compared immediate and deferred antiepileptic drug treatment after a first unprovoked tonic-clonic seizure. Starting on February 1, 1988, all patients examined in 14 university clinics and hospitals in Italy with a first previously untreated, unprovoked tonic-clonic seizure were considered for recruitment. Some FIRST participants were followed up for ten years although the data described here includes only six years of follow-up.

Table 1 provides a demographic summary of people in MESS, NGPSE, FIRST and WA. Patients with missing outcome data (no data following index seizure) have been excluded – for this reason Table 1 summarises 620 patients within the MESS dataset rather than the 637 summarised in the earlier publication [3].

**Table 1:** Demographics of all analysed participants: bold entries relate to variables included in the MESS multivariable model.

		MESS	NGPSE	WA	FIRST
Characteristic		(n = 620)	(n = 274)	(n = 847)	(n = 305)
Age at firstseizure in years					
	Median(IQR)	33·0 (21·9, 49·9)	50·3 (31·8, 68·8)	39·0 (26·0, 56·0)	28·0 (20·0, 46·0)
Gender					
	Male	404 (65)	135 (49)	540 (64)	173 (57)
**Aetiology**					
	**Remote** **symptomatic**	**99 (16)**	**156 (57)**	**270 (32)**	**22 (7)**
**Epilepsy in first** **degree relative**					
	**Yes**	**67 (11)**	**21 (8)**	**93 (11)**	**36 (12)**
	**No**	**553 (89)**	**253 (92)**	**730 (86)**	**269 (88)**
	**Missing**	**-**	**-**	**24 (3)**	**-**
**First seizure** **occurred from** **sleep**					
	**Yes**	**109 (18)**	**40 (15)**	**202 (24)**	**NA**
	**No**	**510 (82)**	**234 (85)**	**643 (76)**	**NA**
	**Missing**	**1 (0)**	**-**	**2 (0)**	**NA**
**EEG results**					
	**Normal**	**278 (45)**	**50 (18)**	**420 (50)**	**144 (47)**
	**Abnormal**	**304 (49)**	**71 (26)**	**405 (48)**	**161 (53)**
	**Not clinically** **indicated**	**38 (6)**	**153 (56)**	**22 (2)**	**-**
**CT/MRI scan results**					
	**Normal**	**444 (72)**	**57 (21)**	**541 (64)**	**246 (81)**
	**Abnormal**	**72 (11)**	**39 (14)**	**240 (28)**	**59 (19)**
	**Not clinically** **indicated**	**104 (17)**	**178 (65)**	**66 (8)**	**-**
**Treatment Policy**					
	**Immediate/On** **Treatment**	**307 (50)**	**78 (28)**	**233 (28)**	**156 (51)**

Entries are number (%) unless otherwise stated.

NA = Not available.

In all of the included studies, when treatment was given, the clinician chose the drug they considered optimum for the individual. In MESS, people who were randomised to treatment were given carbamazepine (46%), valproate (46%), phenytoin (3%), lamotrigine (3%), or another drug (2%). In NGPSE, many AEDs were prescribed but the most common were phenytoin (37% of all treated participants), carbamazepine (36%), and sodium valproate (19%). In WA, initial AED selection included valproate (36%), phenytoin (47%), and carbamazepine (12%). In FIRST, of those who were treated, the most common AEDs prescribed were phenobarbital (50%), carbamazepine (30%), sodium valproate (16%), and phenytoin (5%).

### External Validation

A review of studies [11] that externally validated a prognostic model over the last ten years found that the most frequently implemented methods of external validation were discrimination (64 of 109 studies) and calibration (23 of 109 studies) and we employed these methods. (Full details of the literature review are available on request). We examined discrimination via Harrell’s c-index [12] and calibration via calibration plots [13]. We also considered discrimination and calibration via Kaplan-Meier curves and hazard ratios for risk groups [14].

Discrimination is the ability of a model to allocate people who experience the event of interest a higher predicted probability of experiencing the event than that allocated to those who did not experience the event. We assessed discrimination via Harrell’s *c*-index [12]. This measures the proportion of all possible patient pairs – all possible combinations of patients where one patient has the event and the other does not, and the patient with the event has the shorter follow-up time - in which the predictions and outcomes are concordant. If the predicted time free of the event is longer for the subject who did not experience the event, the predictions for that pair are concordant with the outcomes. A value of *c* of 0.5 corresponds to the concordance expected by chance and 1 corresponds to perfect concordance. For example, a *c*-statistic of 0.6 would mean that, for a random pair of patients, the probability of the patient who had a second seizure first having the shorter predicted probability of a second seizure is 60%. It is calculated using the ‘coxph’ package with R.

Kaplan-Meier curves for risk groups can be used in assessing both model discrimination and calibration. The more widely separated the curves, the better the discrimination. Discrimination can also be compared between datasets by visually comparing the Kaplan-Meier curves for the risk groups. Additionally, if two survival curves are more widely separated the hazard ratios for the groups tend to be larger.

Calibration describes how well the estimates of risk from the model correspond to the risk from the observed data [13] and can be described as a measure of the extent of bias in a model. A model is well calibrated when predicted and observed probabilities of an event agree. We assessed calibration by plotting the observed probabilities of a second seizure against predicted probabilities of the event based on the fitted model. If the model was perfectly calibrated, the predicted and observed values would rest on a 45 degree line – the predicted probability of a second seizure for a patient would be identical to their observed probability of a second seizure [14]. The plots were created using the ‘val.surv’ function within R. Good calibration may also be inferred if the survival curves for a given risk group agree well between the development and validation datasets [14].

### Data Analysis

In the original MESS model and the work presented here, we included people aged 16 years or over on the day of their first seizure as this population are most relevant to driving. The outcome, time from first to second seizure, was calculated for each individual with observations censored at date of last follow-up if a second seizure was not experienced.

Model discrimination and calibration were examined for the Cox proportional hazards model fitted to the MESS data and also for the equivalent model fitted to the validation datasets in turn. The difference in the concordance between the development and validation dataset was then calculated. If the differences were similar (informally assessed; ≤0.05) it was reasonable to conclude that the model was externally valid. Additionally, we created risk groups for MESS by categorising the prognostic index into four groups using cut-points on the prognostic index determined by Cox’s method which minimises the loss of information that occurs with grouping [15]. The required cut-points are the 16^th^, 50^th^ and 84^th^ centiles, giving two smaller groups at relatively low and high risk of recurrence and two larger groups at lower and higher intermediate risks [14]. These risk groups were fitted to each dataset (MESS, NGPSE, WA and each type of imputed FIRST dataset), plotted, and associated hazard ratios were calculated. We also produced a Kaplan-Meier curve for the low risk group in each dataset and visually determined calibration by comparing the curves for NGPSE, WA and FIRST to the curve for MESS.

‘Seizures only while asleep’ was missing completely from FIRST. Therefore five methods of handling a missing covariate were applied when assessing external validation via the FIRST dataset: (1) variable matching – refit the model with the development dataset restricted to include only covariates that are available in the validation dataset; (2) random selection with replacement – each entry of the missing variable is imputed by randomly selecting an entry from the equivalent variable in the development dataset; (3) single imputation via estimated proportions – each entry of the missing variable is imputed with a random number based on the summary statistic(s) of the equivalent variable in the development dataset; (4) hot deck imputation – each entry of the missing variable is imputed with values recorded for similar respondents in the development data; and (5) random selection with replacement multiple times – values of the missing covariate from the development set are randomly selected, with replacement, to produce a number of datasets which are averaged to produce an imputed covariate.

Handling missing data in these ways may lead to biased estimates or data that do not reflect the sampling variability and marginal distributions which could lead to distorted associations [16]. However, simply ignoring the missing variable would be more problematic as it would prevent inclusion of FIRST for model validation.

Covariate values missing for some participants were excluded from these analyses. In FIRST, unlike the other studies, all participants had an EEG and CT/MRI hence there was no ‘not clinically indicated’ category. Following a sensitivity analysis, the EEG and CT/MRI variables were collapsed to two categories each in MESS (normal or abnormal) to match FIRST for the external validation of MESS via FIRST only.

### Fitting of Model to Super-population

Having shown that the model developed using the MESS data generalised fairly well to the NGPSE, WA and FIRST datasets, we fitted the MESS prognostic model to a combined dataset (super-population) of all four datasets to further improve generalisability and precision. The model was stratified by study and the missing sleep variable in FIRST was treated as missing data within the sleep covariate for this super-population model.

Given the issue with the ‘sleep’ variable in FIRST we also performed a sensitivity analysis which excluded FIRST from the pooled analysis. Additionally we performed a sensitivity analysis which excluded WA from the pooled analysis as it showed the poorest validation of the MESS model.

The probability of someone who is seizure free at six months after the index seizure, remaining seizure free throughout months seven to 18 was calculated by dividing the probability of being seizure free for 18 months by the probability of being seizure free for six months. This can be interpreted as the relative probability of being seizure free to 18 months if six months seizure freedom has already been achieved. Conditional probabilities for other time points were calculated similarly, and the confidence intervals for these estimates were calculated utilising a revised version of Greenwood’s formula [17]–[19].

To determine annual recurrence risks for combinations of risk factors the baseline survivor function was estimated from the multivariable model assuming a piecewise linear assumption. The estimate was subsequently raised to a suitable power calculated from combinations of variable coefficient estimates [20]. From this, conditional probabilities can be calculated in the manner described above.

Additionally, the seizure recurrence risk in the next 12 months for any combination of risk factors can be calculated for each time point. Consequently the time point where the seizure recurrence risk in the next 12 months falls below 20% can be determined – the risk threshold for returning to drive.

## Results

**Figure 1:**
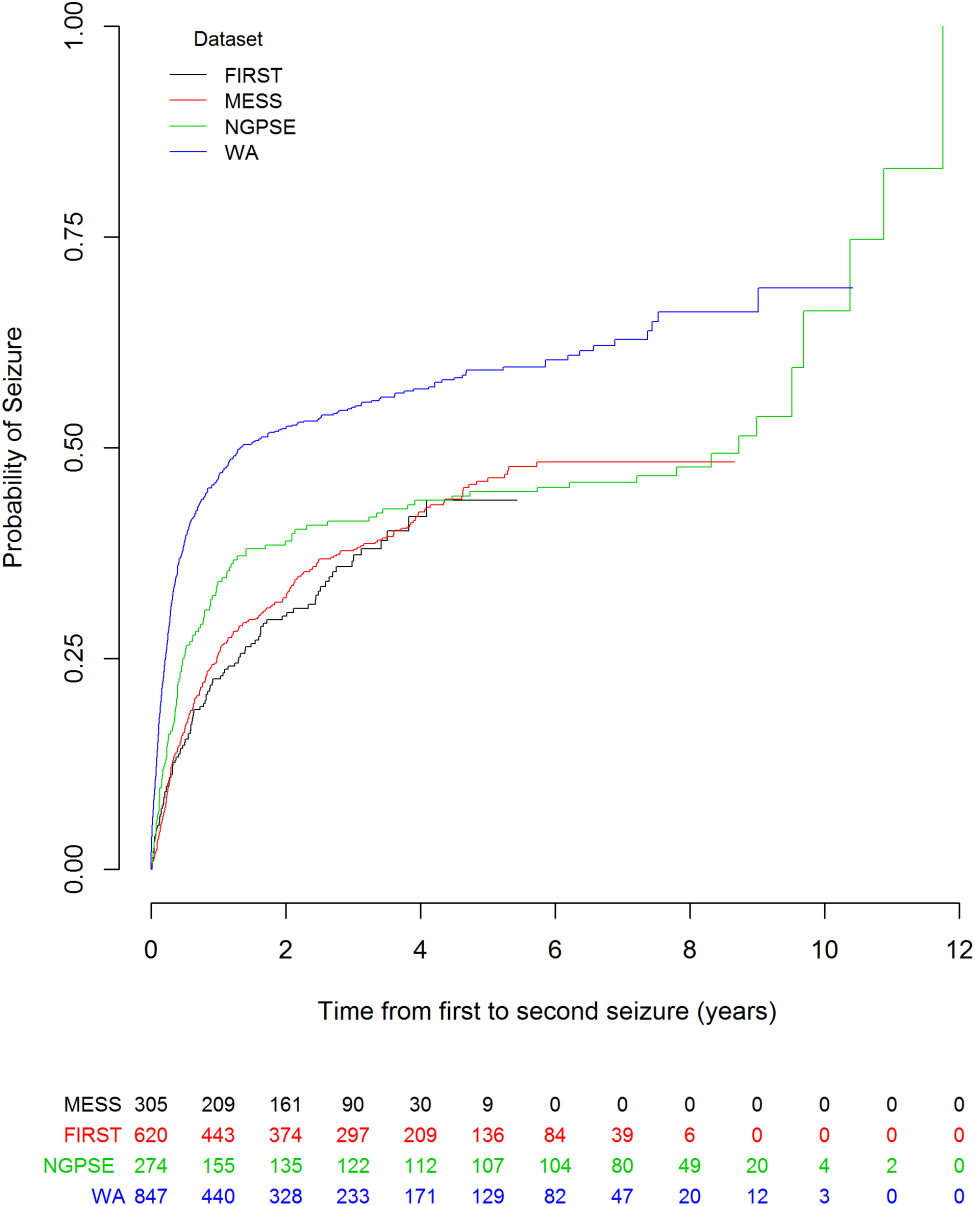
Kaplan-Meier curve for time from first to second seizure for MESS, NGPSE, WA and FIRST with numbers at risk.

As might be expected, there are some differences in participant characteristics across the datasets. People in NGPSE tended to be older than those in MESS and there were more females in NGPSE, although the variables for age and gender were not included in the multivariable model validated. Considerably more people in NGPSE were classified as having a remote symptomatic aetiology than those in MESS while FIRST had a lower rate suggesting that there may be systematic differences in the way this was classified across studies. In the NGPSE cohort there were more results for EEG and CT/MRI scan that were not clinically indicated, in part reflecting the era in which the study was undertaken. Characteristics for people in the WA dataset were mostly similar to those in MESS. The Kaplan-Meier curve for these datasets, Figure 1, shows WA also had a higher risk of seizure.

The FIRST dataset had a similar distribution of characteristics to those of MESS. Patients in FIRST, that were available for this analysis, were followed-up for a considerably shorter duration than those in MESS.

Despite the differences in characteristics and follow-up it is plausible that the NGPSE, FIRST and WA datasets came from the same ‘super-population’ as MESS. This is because all four datasets consider patients with a first unprovoked seizure and consequently can be considered to come from the same super-population of patients.

FIRST is the closest match to MESS in terms of the proportions of patients with certain characteristics, however, the follow-up is shorter and a covariate that was significant in the model is missing (seizures while asleep). In the case of NGPSE and WA, information on the same number of covariates is available but the proportions of patients with some characteristics are not always similar to the MESS data, in particular, the proportion of people with remote symptomatic aetiology in NGPSE. However, this again represented clinical practice where, in many cases, the only similarity between patients is that they have had a seizure.

### External Validation Results

**Figure 2:**
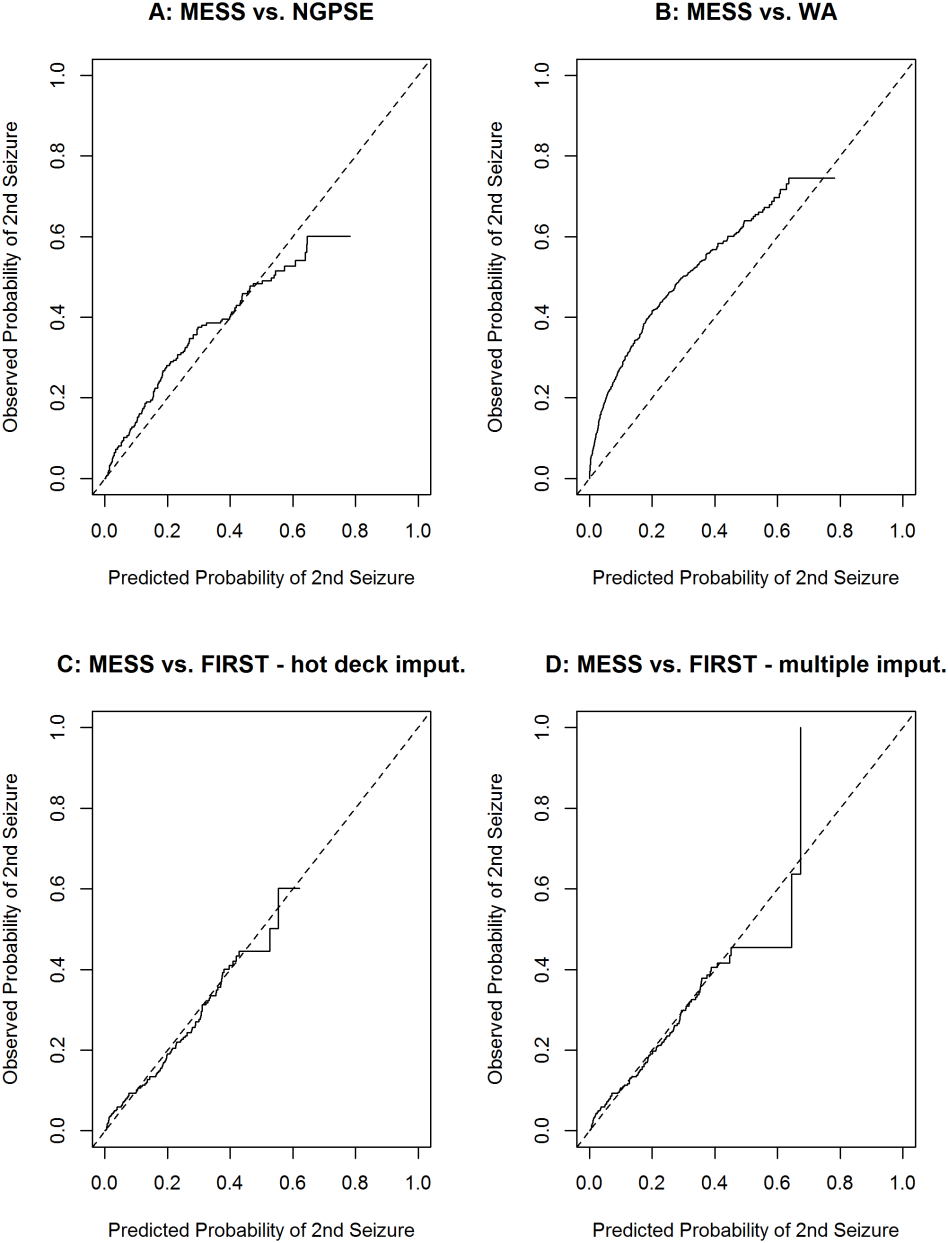
Calibration plots for MESS compared to NGPSE (A), WA (B), FIRST - variable matching (C) and FIRST - hot deck imputation (D).

Figure 2 shows calibration plots for external validation of MESS with NGPSE (Figure 2A), WA (Figure 2B) and FIRST (Figure 2C & 2D). Only plots for variable matching (Figure 2C) and hot deck imputation (Figure 2D) methods are shown for FIRST but plots for the other three methods of handling a missing covariate are similar. NGPSE appears to validate quite well while the WA dataset displays the poorest external validation. The FIRST data displays the best calibration plot – the data fits very well along the 45 degree line. This may be because of the imputation which makes the dataset artificially related to MESS. Therefore, in a sensitivity analysis, we also fitted calibration plots with the model that excludes the sleep variable. The plots were very similar to those shown in Figure 2 (not shown here but available on request).

Concordance estimates and confidence intervals (Table 2) for NGPSE and WA are very similar to those of MESS, suggesting that the MESS model generalises well. Confidence intervals for the concordance statistics using the FIRST data are fairly similar across all imputation methods. It is likely that the *c*-index for the FIRST dataset is higher than in the original MESS study due to the case mix – FIRST is a more homogenous population as it includes patients with only a first tonic-clonic seizure while MESS also included patients with other seizure types [21]. Together with the relatively small differences in concordance between MESS and the imputed FIRST datasets, the MESS model seems to generalise fairly well to the FIRST data too.

**Table 2:** Summary of discrimination measure for MESS, NGPSE, WA and FIRST.

Dataset andmodifications	Concordance (*c*)statistic (95%Confidence Interval)	Difference inConcordancestatistic
MESS	0·59 (0·56, 0·63)	NA
NGPSE	0·60 (0·55, 0·65)	0·01
WA	0·59 (0·56, 0·62)	0·00
MESS: No Sleep Variable*	0·58 (0·54, 0·61)	NA
FIRST: Variable Matching	0·65 (0·59, 0·70)	0·07
MESS: Sleep Variable**	0·59 (0·55, 0·62)	NA
FIRST: Random Selection	0·65 (0·60, 0·70)	0·06
FIRST: Single Imputation	0·65 (0·60, 0·71)	0·06
FIRST: Hot Deck	0·66 (0·60, 0·71)	0·07
FIRST: Multiple Imputation	0·65 (0·60, 0·70)	0·06

*Sleep variable removed to match variables available in the FIRST dataset; EEG & CT/MRI scan result reduced to two categories.

**Sleep variable re-included; EEG & CT/MRI result reduced to two categories.

NA = Not applicable.

**Figure 3:**
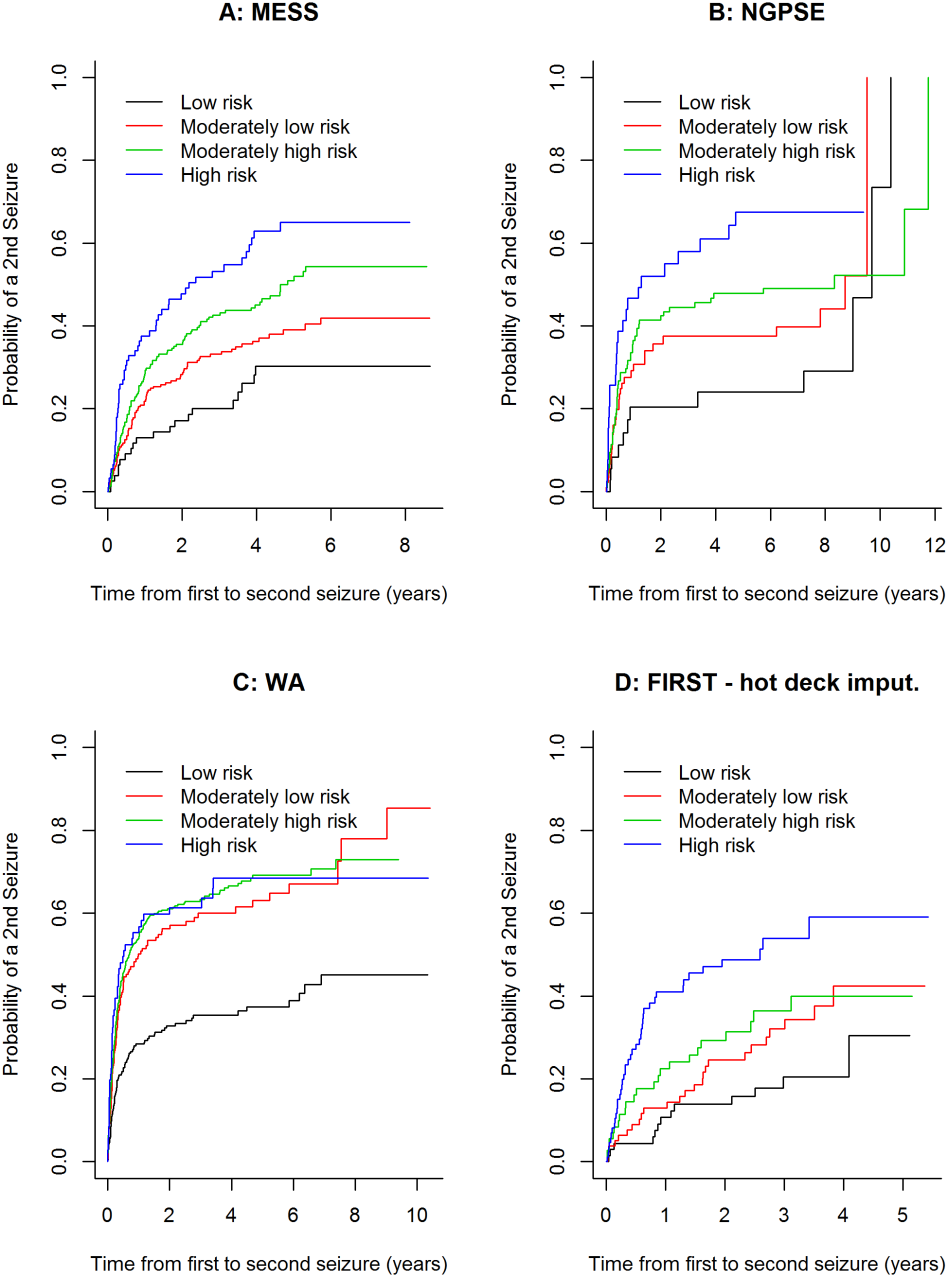
Kaplan-Meier curves for risk groups in MESS (A) fitted to NGPSE (B), WA (C) and FIRST – hot deck imputation (D).

The Kaplan-Meier curve for the MESS risk groups can be seen in Figure 3 (Figure 3A). Kaplan-Meier curves for the fitting of these risk groups to NGPSE (Figure 3B), WA (Figure 3C) and FIRST (Figure 3D) can also be seen. Only the results for the hot-deck imputation of the sleep variable in FIRST are shown, but plots of the other four imputation methods are similar. MESS, NGPSE and FIRST display well separated curves suggesting good discrimination. WA on the other hand displays poor separation and consequently poor discrimination.

The hazard ratios for the risk groups can be seen in Table 3. The results for NGPSE and FIRST are similar to the MESS results, irrespective of the imputation method used for the FIRST dataset. This confirms our conclusion of good model discrimination. The hazard ratios for WA are not very comparable which suggests poor discrimination.

**Table 3:** Hazard ratios for risk groups in MESS fitted to NGPSE, WA and FIRST.

	Risk Group Comparison		
	Hazard Ratio (95% Confidence Interval)		
Dataset	Moderately Lowvs. Low	Moderately Highvs. Low	High vs. Low
MESS	1·54 (0·95, 2·52)	2·12 (1·31, 3·42)	3·11 (1·86, 5·22)
NGPSE	1·48 (0·77, 2·89)	1·67 (0·89, 3·14)	2·82 (1·41, 5·63)
WA	2·11 (1·58, 2·82)	2·34 (1·83, 3·00)	2·46 (1·72, 3·52)
MESS: No Sleep Variable*	1·62 (1·09, 2·40)	2·02 (1·40, 2·93)	3·00 (2·02, 4·44)
FIRST: Variable Matching	1·50 (0·71, 3·17)	1·84 (1·06, 3·18)	2·71 (1·68, 4·36)
MESS: Sleep variable**	1·54 (0·95, 2·52)	2·12 (1·31, 3·42)	3·11 (1·86, 5·22)
FIRST: Random Selection	1·35 (0·71, 2·58)	1·94 (1·05, 3·60)	3·06 (1·73, 5·41)
FIRST: Single Imputation	1·62 (0·82, 3·20)	2·33 (1·20, 4·54)	3·78 (2·02, 7·07)
FIRST: Hot Deck	1·74 (0·89, 3·41)	2·10 (1·06, 4·15)	3·85 (2·06, 7·19)
FIRST: Multiple Imputation	1·74 (0·89, 3·41)	2·18 (1·11, 4·29)	3·77 (2·02, 7·06)

*Sleep variable removed to match variables available in the FIRST dataset; EEG & CT/MRI scan result reduced to two categories.

**Sleep variable re-included; EEG & CT/MRI result reduced to two categories.

NA = Not applicable.

**Figure 4:**
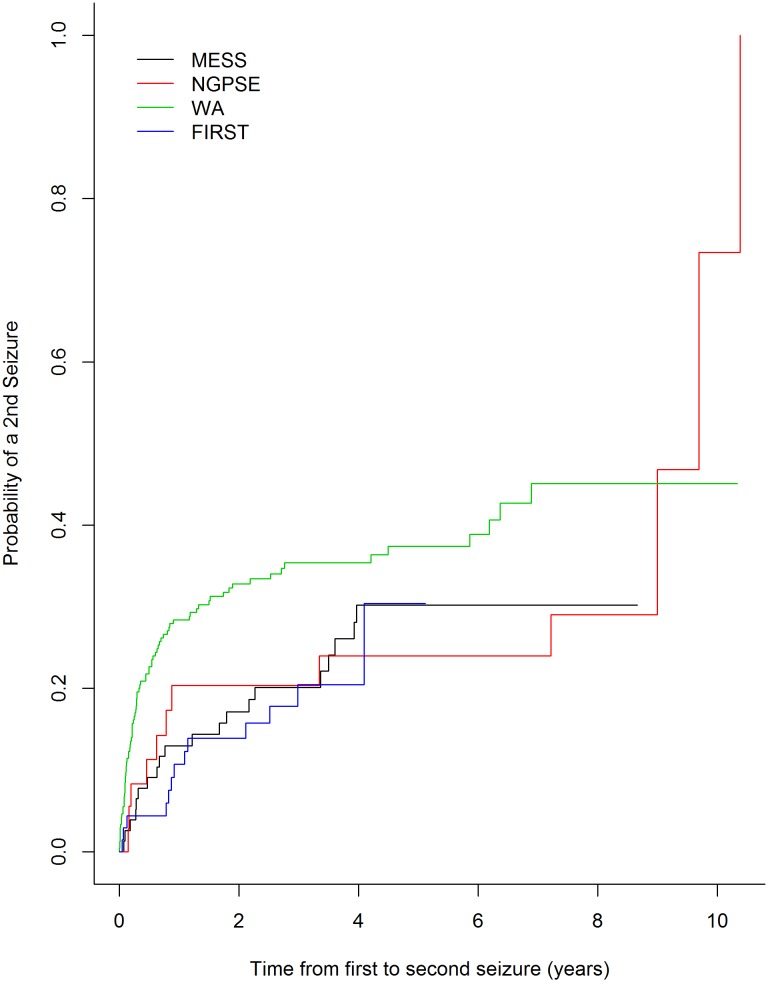
Kaplan-Meier curve for low risk patients in MESS, NGPSE, WA and FIRST – hot deck imputation.

As shown in Figure 4, survival curves for the low risk group agree well between MESS (development dataset) and NGPSE as well as between MESS and FIRST – only hot deck imputation results are shown here but results for the other methods of imputation are very similar. This infers good calibration in these cases. The agreement is not so good for WA confirming our calibration plot which showed poor calibration for WA. Additionally, from these plots, we can see that low risk patients have a less than 20% risk of a second seizure for about two years after their index seizure.

### Refitting of Model to ‘Super-population’

Having shown that the model developed using the MESS data generalises fairly well, we proceeded to fit the model in a combined dataset (super-population) of MESS, NGPSE, WA and FIRST, stratified by study, to improve generalisability and precision.

Effect estimates from the multivariable models fitted in the two sensitivity analyses can be seen in Table S1. The first sensitivity analysis excluded FIRST from the pooled analysis because of the missing ‘sleep’ variable in FIRST. The second excluded WA as it showed the poorest validation of MESS. The results were broadly similar to those for the pooled analysis including all four datasets.

Effect estimates from the multivariable model from both MESS and the super-model can be seen in Table 4. The estimates are very similar to those from the MESS model and, as expected, the confidence intervals are narrower due to the additional data included.

**Table 4:** Effect estimates from the multivariable model – MESS multivariable model fitted to super-population comprising MESS, NGPSE, WA and FIRST.

		Hazard Ratio (95% CI)	
Covariate		MESS	Super-population
Cause of seizure			
	Not remote symptomatic	1.00	1.00
	Remote symptomatic	1.33 (0.95, 1.87)	1.36 (1.15, 1.62)*
Epilepsy in first degree relative			
	No	1.00	1.00
	Yes	1.33 (0.94, 1.90)	1.32 (1.09, 1.60)*
Seizures only while asleep			
	No	1.00	1.00
	Yes	1.47 (1.09, 1.97)*	1.31 (1.12, 1.53)*
EEG results			
	Normal	1.00	1.00
	Abnormal	1.55 (1.20, 2.01)*	1.48 (1.29, 1.71)*
	Not clinically indicated	1.29 (0.74, 2.27)	1.12 (0.83, 1.51)
CT or MRI scan results			
	Normal	1.00	1.00
	Abnormal	1.07 (0.72, 1.61)	1.08 (0.89, 1.29)
	Not clinically indicated	1.29 (0.94, 1.78)	1.00 (0.81, 1.23)
Treatment policy			
	Delayed	1.00	1.00
	Immediate	0.82 (0.64, 1.05)	0.85 (0.74, 0.98)*

HR>1 implies risk of second seizure is greater in alternative category than in the baseline category.

*Significant values (p<0.05).

Table 5 shows the risk of seizure recurrence over 12 months for a range of periods of seizure freedom following a first seizure, estimated from the super-population model. The risk of a seizure in the next 12 months is significantly below the 20% risk threshold set by the Driving and Vehicle Licensing Agency (DVLA) after six months for people who were treated immediately. The point estimate for people who did not start treatment is below 20% but the upper bound of the confidence interval is 21%.

**Table 5:** Risk of seizure recurrence over 12 months at time points after first seizure: risk (%, 95% confidence interval) – unadjusted estimates obtained from super-population comprising MESS, NGPSE, WA and FIRST.

	Immediate Treatment		Delayed Treatment	
Time after first seizure(months)	No. at riskof seizure	Risk of seizure innext 12 months (%)	No. at riskof seizure	Risk of seizure innext 12 months (%)
6	580	15 (12 to 18)	872	18 (15 to 21)
12	500	8 (6 to 11)	748	10 (8 to 13)
18	451	6 (4 to 9)	657	9 (7 to 11)
24	414	7 (4 to 9)	585	7 (5 to 9)

**Figure 5:**
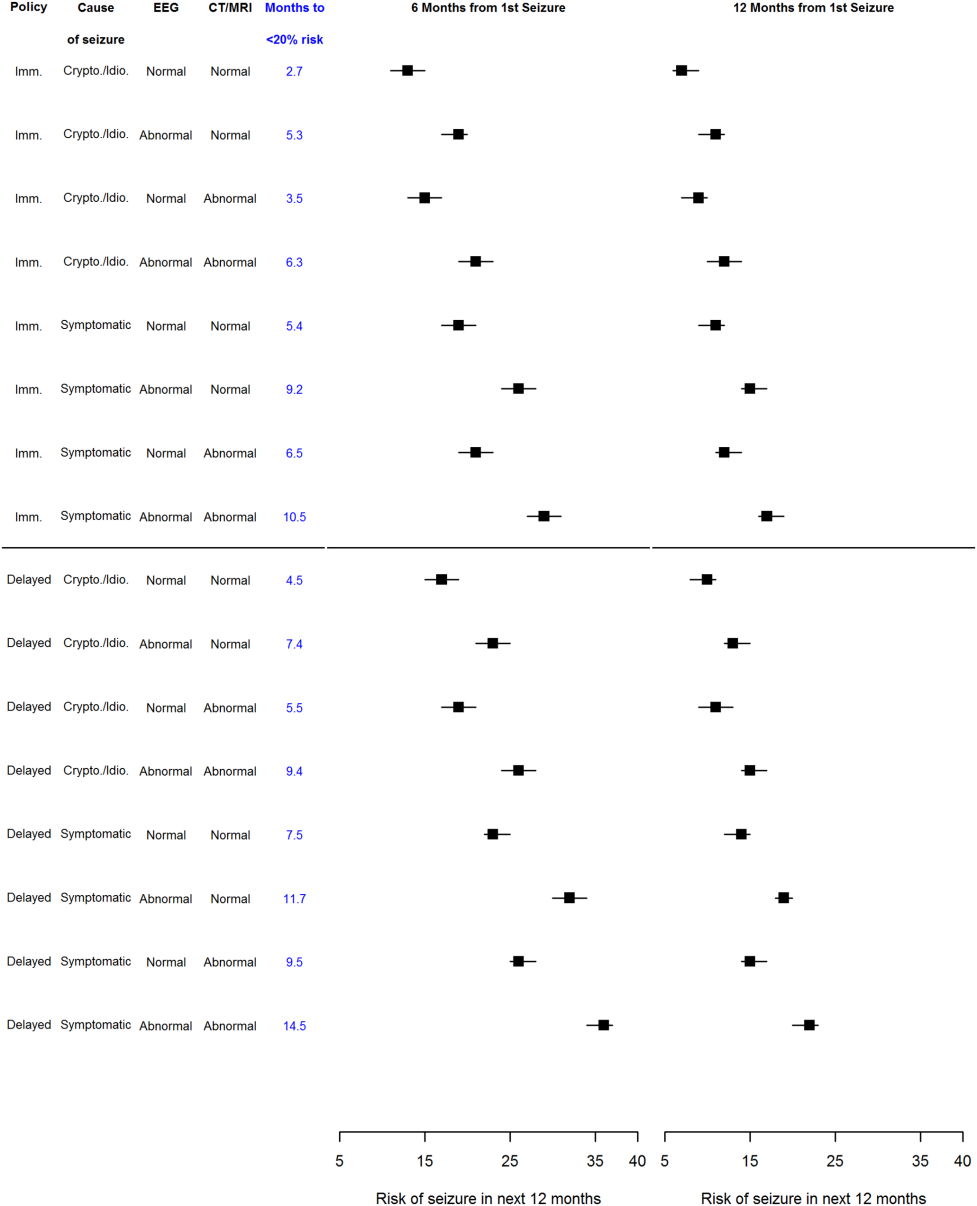
Risk of seizure recurrence in next 12 months estimated from multivariable model at specific seizure-free periods. Estimates presented assume seizures not confined to sleep and no first degree relative with epilepsy - MESS multivariable model fitted to super-population comprising MESS, NGPSE, WA and FIRST. Imm = Immediate; Crypto,/Idio/ = Cryptogenic/Idiopathic; Symptomatic = Remote symptomatic. Risks are risk of seizure in next 12 months with associated 95% confidence interval.

Figure 5 shows the risk of seizure recurrence in the next 12 months for differing groups estimated from the super-population multivariable model. These estimates are conditional on the individual being recurrence free at six and 12 months following a first seizure. These results assume seizures are not confined to sleep and that there are no first degree relatives with epilepsy. The figure also shows (in blue) the time point at which the estimate for seizure recurrence drops below 20%.

Of note are the results for people with an idiopathic or cryptogenic seizure with a normal CT/MRI and an abnormal EEG who do not start AED treatment; at six months following a first seizure this group has a recurrence risk significantly above 20%, although the estimate falls below 20% at 7.4 months. Similarly, for those with a remote symptomatic seizure and an abnormal EEG but normal CT/MRI, the recurrence risk does not drop below 20% until 9.2 months if AED treatment is started and 11.7 months if it is not. An abnormal CT/MRI has a smaller impact than EEG on recurrence risk. At six months after a first seizure the 12 month recurrence risk estimates are below 20% for people with a cryptogenic/idiopathic seizure, an abnormal CT/MRI, and a normal EEG whether they start AED treatment or not.

## Discussion

We have investigated the external validity of a prognostic model for risk of a second seizure following a first ever seizure via the NGPSE, WA and FIRST datasets. Following fair external validation of the MESS model using NGPSE, WA and FIRST, we fitted the MESS model to a super-population comprising all four datasets. These data can further inform driving regulations and the current DVLA guidelines [22] that state, following a first unprovoked seizure, a person must refrain from driving for six months unless there are clinical factors or investigation results which suggest an unacceptably high risk of a further seizure, i.e. 20% or greater per annum, in which case the time off driving is increased.

### Model Validation

The MESS model appears to be externally valid, to different degrees, using different datasets although with respect to individual studies, the discrimination and calibration measures appear to give rather discrepant conclusions. The model generalises fairly well to the NGPSE and WA datasets and not quite so well to the FIRST dataset according to the *c*-statistic while it generalises very well to FIRST, quite well to NGPSE and poorly to WA according to the calibration plots. According to the Kaplan-Meier curves for the risk groups and the associated hazard ratios, the model generalised fairly well to NGPSE and FIRST but poorly to WA, conclusions also reached by visual inspection of the curves for low risk patients.

The differences in discrimination could be because of the heterogeneity in EEG and CT/MRI scan results – results were classified differently in FIRST than in MESS which may explain why the model discrimination was poorer for FIRST according to the *c*-statistic. The differences in calibration may be because of the varying risks of seizure recurrence across the studies – patients in WA tended to have a higher risk of a seizure recurrence than those in MESS and patients in NGPSE also showed a slightly higher risk of seizure recurrence in the four years after recruitment than MESS.

### Clinical Messages

The combined studies have over 2,000 people, more than three times the number in MESS. Therefore the estimates derived from the model are more accurate with smaller confidence intervals. These results based on a super-population of people with a first unprovoked seizure, provide further evidence that, in general, the risk of seizure recurrence following a first ever seizure falls below 20% by six months irrespective of the treatment policy. Subgroups at higher risk can, however, be identified. If a decision is made not to start AED treatment, all those with a remote symptomatic seizure have a significantly greater than 20% recurrence risk at six months following a first seizure, while if treatment is started those with a remote symptomatic seizure and a normal EEG do not. If a decision is made not to start treatment for people with a cryptogenic/idiopathic seizure, those with an abnormal EEG have a significantly greater than 20% recurrence risk at six months following a first seizure, while if treatment is started they do not.

Driving regulators will need to take these results into account when deciding driving policy and in particular whether to attempt to stratify risk in their guidance. The decisions made by regulators could influence decisions about starting treatment, especially for higher risk groups who might be able to return to driving sooner if they started antiepileptic drug treatment. Clinicians will need to take account of these results when deciding which investigations are required and when counselling patients about starting treatment. The prognostic importance of an abnormal EEG may be challenging, as patients who have this finding may be disadvantaged compared to patients that do not have an EEG. One could argue that this should be communicated with patients before an EEG is requested.

### Limitations

The study has certain methodological limitations. Firstly, we have not fitted interaction terms in the multivariable model. Additionally, only four of several possible methods of external validation have been considered and the methods do not lead to the same conclusions. In addition, rather than a clear-cut answer, different degrees of external validation are likely and the degree of ‘acceptable’ external validity may be influenced by what the model will be used for as the consequences of a poorly validated model in practice may have very different impacts.

Data used to externally validate a prognostic model should come from the same super-population. There are, however, no guidelines suggesting how to check whether the development and validation datasets are from a super-population. The three external validation datasets investigated here were plausibly related datasets to MESS but were not a perfect match. These differences are, however, likely to have a minimal statistical impact on the validity of using the non-matched data for external validation purposes. This is because external validation focuses on showing that a prognostic model or index, developed in one dataset, also fits different data – if the data were too statistically similar, the model may not be generalisable more widely.

Given that the model appears to be externally valid in populations that are different in some respects to the MESS dataset it may be possible to conclude that the model is more useful as a clinical tool. The differences in populations may actually be considered as an advantage too as they suggest that our model is valid in a variety of settings.

Similarly, the model being externally validated is for use in the UK. Two out of the three external validation datasets were, however, not collected in the UK. Driving regulations in the EU, and further afield, currently differ among member states but minimum standards for driving are now in the process of being implemented. In Australia a person may regain a driving license following a first seizure provided they have been seizure free for at least six months [23] which is in line with the UK [24] and EU guidelines [25]. Therefore, by showing that our model is externally valid in datasets from around the world, that differ in design and included drugs for example, there is evidence to support one overall prognostic model for risk of second seizure following a first. This would ensure global harmonisation of driving regulations.

The choice of a 20% standard is arbitrary, although this standard has been generally accepted across the EU during a process of regulation harmonization. If the standard were changed, data from these analyses could be extended to inform regulators, clinicians and the public.

### Conclusions

Our prognostic model has been validated, and extended to include additional data to improve precision of estimates. The model is a valuable tool for predicting risk of seizure recurrence following a first seizure for people with various combinations of risk factors. It can be used to inform driving regulation both within the UK and across the world.

## Supporting Information

Table S1 **Effect estimates from the multivariable models considered in the sensitivity analyses – MESS multivariable model fitted to super-population comprising MESS, NGPSE, WA and FIRST compared to super-population comprising MESS, NGPSE and WA and super-population comprising MESS, NGPSE and FIRST.**
(DOCX)
