# Correction: External Validation of a Prognostic Model for Seizure Recurrence Following a First Unprovoked Seizure and Implications for Driving

**DOI:** 10.1371/journal.pone.0113327

**Published:** 2014-11-06

**Authors:** The PLOS ONE Staff

There is an error in Figure 1, “Kaplan-Meier curve for time from first to second seizure for MESS, NGPSE, WA and FIRST with numbers at risk.” Please see the corrected Figure 1 here.

**Figure 1:**
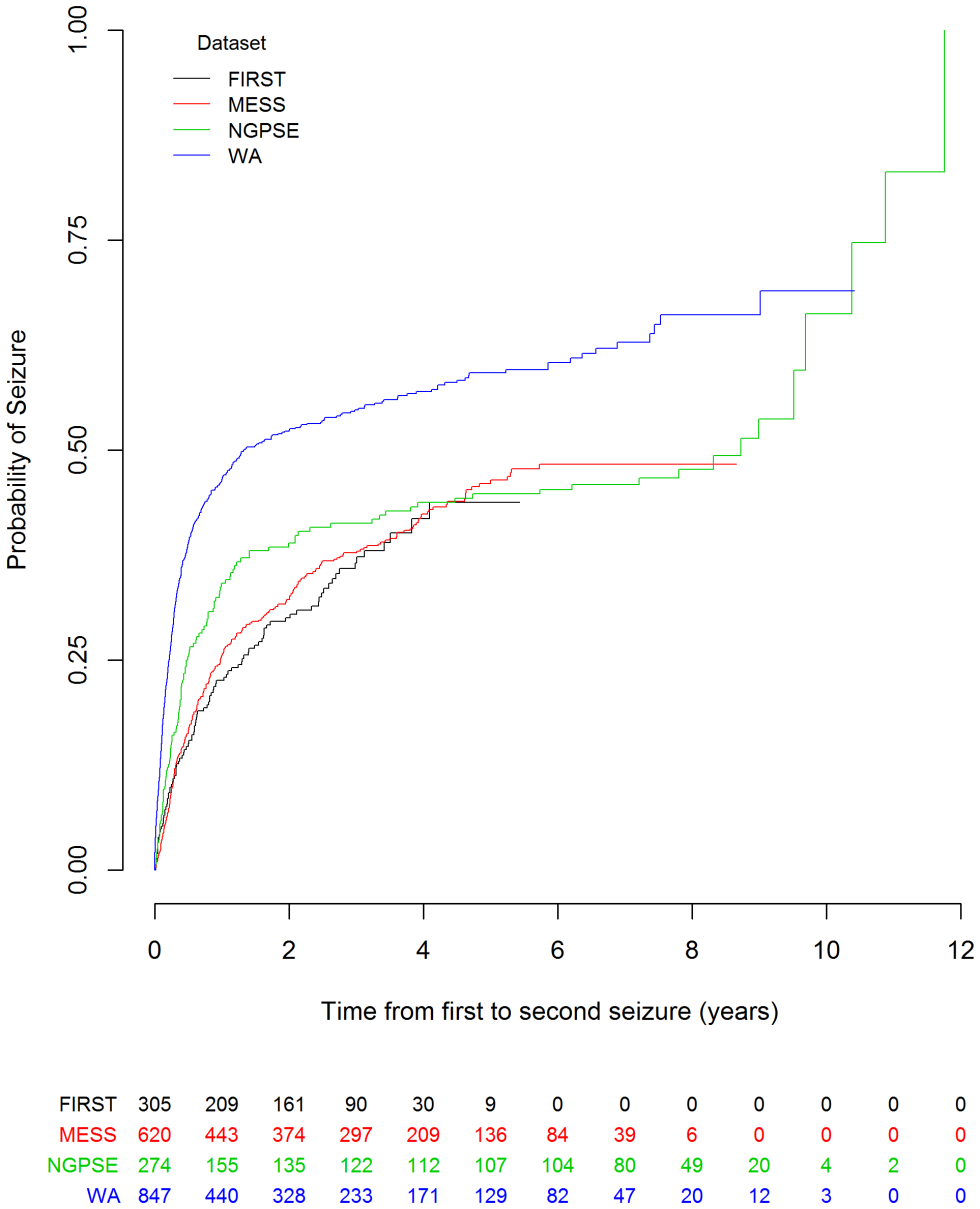
Kaplan-Meier curve for time from first to second seizure for MESS, NGPSE, WA and FIRST with numbers at risk.

## References

[1] Bonnett LJ, Marson AG, Johnson A, Kim L, Sander JW, et al (2014) External Validation of a Prognostic Model for Seizure Recurrence Following a First Unprovoked Seizure and Implications for Driving. PLoS ONE 9(6): e99063 doi:10.1371/journal.pone.0099063 24919184 10.1371/journal.pone.0099063 PMC4053525

