# Supplementary material for: External Validation of a Prognostic Model for Seizure Recurrence Following a First Unprovoked Seizure and Implications for Driving
Source: PLoS One. 2014 Jun 11;9(6):e99063. doi: 10.1371/journal.pone.0099063 (PMC4053525; doi:10.1371/journal.pone.0099063)
Supplement: Table S1 — Effect estimates from the multivariable models considered in the sensitivity analyses – MESS multivariable model fitted to super-population comprising MESS, NGPSE, WA and FIRST compared to super-population comprising MESS, NGPSE and WA and super-population comprising MESS, NGPSE and FIRST. (DOCX) [file pone.0099063.s001.docx]

Table S2: Effect estimates from the multivariable models considered in the sensitivity analyses – MESS multivariable model fitted to super-population comprising MESS, NGPSE, WA and FIRST compared to super-population comprising MESS, NGPSE and WA and super-population comprising MESS, NGPSE and FIRST

|  |  | **Hazard Ratio (95% CI) for super-population including the following datasets** | | |
| --- | --- | --- | --- | --- |
| **Covariate** | | **MESS, NGPSE, WA & FIRST (full model)** | **MESS, NGPSE & WA (no FIRST)** | **MESS, NGPSE & FIRST (no WA)** |
| Cause of seizure |  |  |  |  |
|  | Not remote symptomatic | 1.00 | 1.00 | 1.00 |
|  | Remote symptomatic | 1.36 (1.15, 1.62)* | 1.34 (1.12, 1.60)* | 1.30 (1.03, 1.65)* |
| Epilepsy in first degree relative |  |  |  |  |
|  | No | 1.00 | 1.00 | 1.00 |
|  | Yes | 1.32 (1.09, 1.60)* | 1.34 (1.09, 1.64)* | 1.30 (0.99, 1.69) |
| Seizures only while asleep |  |  |  |  |
|  | No | 1.00 | 1.00 | 1.00 |
|  | Yes | 1.31 (1.12, 1.53)* | 1.31 (1.11, 1.53)* | 1.46 (1.14, 1.87)* |
| EEG results |  |  |  |  |
|  | Normal | 1.00 | 1.00 | 1.00 |
|  | Abnormal | 1.48 (1.29, 1.71)* | 1.48 (1.27, 1.71)* | 1.51 (1.24, 1.84)* |
|  | Not clinically indicated | 1.12 (0.83, 1.51) | 1.12 (0.83, 1.52) | 0.88 (0.62, 1.26) |
| CT or MRI scan results |  |  |  |  |
|  | Normal | 1.00 | 1.00 | 1.00 |
|  | Abnormal | 1.08 (0.89, 1.29) | 1.05 (0.86, 1.28) | 1.18 (0.91, 1.54) |
|  | Not clinically indicated | 1.00 (0.81, 1.23) | 0.99 (0.80, 1.22) | 1.11 (0.85, 1.45) |
| Treatment policy |  |  |  |  |
|  | Delayed | 1.00 | 1.00 | 1.00 |
|  | Immediate | 0.85 (0.74, 0.98)* | 0.94 (0.81, 1.09) | 0.75 (0.62, 0.90)* |

* Significant values (p<0.05)
